# A New Organofunctional Ethoxysilane Self-Assembly Monolayer for Promoting Adhesion of Rubber to Aluminum

**DOI:** 10.3390/molecules14104087

**Published:** 2009-10-12

**Authors:** Fang Wang, Juan Xu, Heyi Luo, Jinggang Wang, Qian Wang

**Affiliations:** College of Science, Northwest Agriculture & Forest University, Yangling 712100, China; E-Mail: liurenzu@sohu.com (J.X.)

**Keywords:** aluminum, rubber, self-assembly, monolayer, adhesion

## Abstract

Practical adhesion of rubber to aluminum is measured for various aluminum silanization treatments. In this study, 6-(3-triethoxysilylpropylamino)-1,3,5-triazine-2,4- dithiol (TES) was used as the coupling agent for preparing self-assembly monolayers (SAMs) on an aluminum surface. The structure and chemical composition of the SAMs were analyzed using Fourier transform infra-red spectroscopy (FT-IR) and X-ray photo-electron spectroscopy (XPS). The changes in the surface features of the aluminum surface due to TES treatment were investigated by atomic force microscopy (AFM). The adhesive properties of the silanized aluminum surface and EPDM rubber have been evaluated by a T-peel strength test. The results suggested that the Si-O-Al bonding at aluminum TES interface existed and a TES self-assembly monolayer was formed on the aluminum surface. More than 6.0 KN/m adhesion strength is obtained when the aluminum is silanized with 2.5 mmol/dm^3^ TES, cured at 160 °C and vulcanized with EPDM rubber at 160 °C for 30 min. It is suggested that the TES self-assembly monolayer is bound to aluminum through its ethoxysilyl functional group, and the thiol function group is strongly cross-linked to EPDM rubber, respectively.

## 1. Introduction 

Aluminum alloys are widely used in numerous industrial applications, due to their low cost, light weight, and high mechanical strength. However, the adhesion between polymeric materials and aluminum substrates is usually weak due to poor compatibility of polymers with aluminum surfaces. This poor adherence limits their joint applications. To improve compatibility with polymer materials, adhesion promoters are commonly required.

For many decades, chromate conversion coatings have been reported as the most efficient pre-treatment used as anti-corrosive measures and adhesion promoters on the surface of aluminum employed in the aircraft industry [1,2,3,4,5,6]. The use of chromates is, however, being restricted worldwide, as they are considered highly toxic and carcinogenic [7,8,9,10,11,12,13,14]. Because current environmental legislation is moving towards total exclusion of Cr^6+^ from industrial processes, and due to tightening regulatory pressures in order to reduce hazardous chromium waste, many attempts are being made to develop nontoxic and environmentally friendly alternative methods for promoting adhesion and protecting corrosion of these materials.

Consequently, a totally new method is proposed with the use of silane coupling agents instead of previous technology based on chromate treatment. Silane coupling agents [15,16], which have already been widely used in several surface applications, such as adhesion promoters for metals, polymeric and inorganic materials [17,18,19], appear to be good candidates.

Obviously, this new method requires that the organic alkoxysilane interact with rubber and the metallic surface. The rubber first must be functionalized in order to bind with the silane. Secondly, the metallic surface must be oxidized to provide M-OH groups available to condense with the SiOH groups. Therefore, the metal is coated with an alkoxysilane solution of X-R-Si(OCH_2_CH_3_)_3_ (X being a functional group, i.e., NH_2_, S_8_, SH, etc.), and a condensation reaction occurs between M-OH and the Si-OH groups of the hydrolysed 6-(3-triethoxysilylpropylamino)-1,3,5-triazine-2,4-dithiol, leading to the formation of M–O–Si–R–X after loss of H_2_O. The group X is selected in order to interact with another group Y grafted to the natural rubber (NR–Y), and outstanding adhesion should be expected from the formation of covalent bonds (M–O–Si–R–X–Y–NR) between the two substrates. First experiments following this technology were described by Thiedman *et al*. [20]. Application of this technology for improving the adhesion of brass and zinc tire cords to rubber was proposed later by Van Ooij and co-workers [21] who used bifunctional polysulfide organosilanes and found significant improvements in adherence. It should be noted that, these silane coupling agents were all long-chained silane alkyl hydrosulfide compouds deposited on the metallic substrates, and the metal pretreatments involved conventional methods. To date, the adhesion property of heterocyclic aromatic silane coupling agents was barely been reported.

In this paper, we investigate the formation and characterization of TES self-assembly monolayers on an aluminum surface. The adhesion properties of the modified aluminum substrate to EPDM rubber are studied. The changes in the surface morphology of the aluminum substrate, structure and chemical composition of SAM were evaluated by atomic force microscope (AFM), contact angle, Fourier transform infra-red spectroscopy (FT-IR) and X-ray photoelectron spectroscopy (XPS).

## 2. Results and Discussion 

### 2.1. Wettability (Contact Angle)

**Figure 1:**
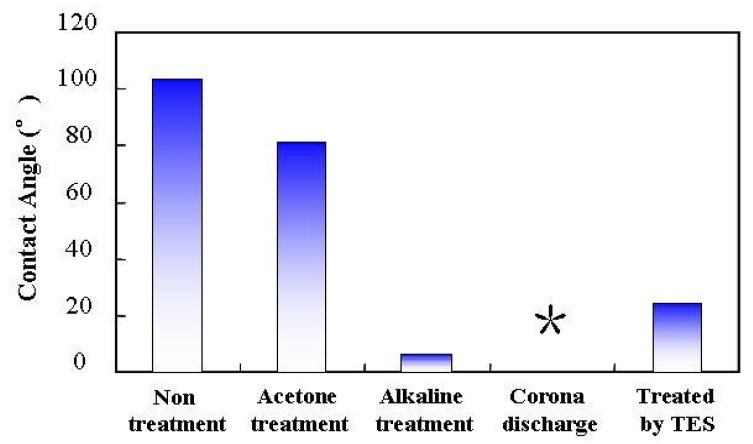
Effect of different pretreatment and modification of aluminum surface on the contact angle.

Figure 1 shows the variation in contact angle of an aluminum surface treated by a series of different procedures. The contact angle of untreated aluminum surface is about 104.6°. In this condition, the aluminum surface is more hydrophobic due to the lack of polar functional groups and has low surface energy. Therefore, the formation of a TES self-assembly monolayer on a non-treated aluminum surface is very difficult. The contact angles of an aluminum surface dramatically decreased after acetone treatment, alkaline degreasing, and corona discharge. The decreases in contact angle indicate the morphology changes and formation of hydrophilic polar groups on the pre-treated aluminum surfaces. It could be presumed that the Bayerite Al(OH)_3_ and Boehmite AlOOH formed on the aluminum surface due to the alkaline pretreatment and corona discharge, improved the wettability of the aluminum surface and make the formation of a TES self-assembly monolayer possible. On the other hand, we observed that the contact angle increased after forming abTES self-assembly monolayer on the aluminum surface, suggesting that a condensation reaction occurred between the polar groups on the treated aluminum surface and the Si-OH groups of prehydrolyzed TES. Thus, a TES self-assembly monolayer is deposited on the aluminum surface. To further confirm the formation of the TES film, FT-IR and XPS measurements were carried out.

### 2.2. Chemical Composition (FT-IR/RAS)

**Figure 2:**
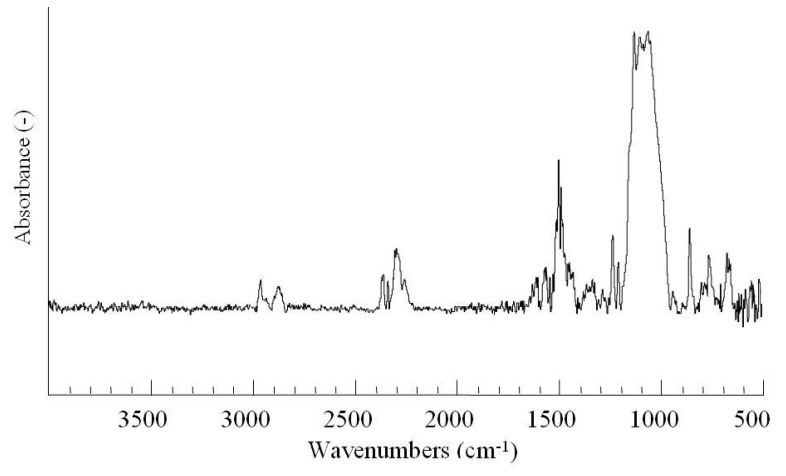
FT-IR spectra of aluminum surface coated by TES self-assembly monolayer (Resolution: 4 cm^−1^; Incident angle: 80°; Scan times: 256).

The structure of TES self-assembly monolayer prepared by dip-coating method was characterized by FT-IR spectroscopy. Figure 2 shows the typical IR spectrum of TES self-assembly monolayer on an aluminum surface. The tiny peak at 2,966 cm^−1^ is due to -CH_3_ asymmetric stretching in -OCH_3_ groups. The peaks at 1,593 cm^−1^ and 1,508 cm^−1^ are attributed to C=S and C=N stretching of the triazine ring in the TES self-assembly monolayer. A sharp peak at 1,258 cm^−1^ due to the -Si-C symmetric bending in –Si(CH_2_)_2_– groups is also observed. A broad peak between 1,161 cm^−1^ and 1,086 cm^−1^ corresponding to -Si-O and Al-O- asymmetric stretching in -Si-O-Al and Al-O-O-H, is clearly observed. It indicates that Boehmite layers have formed on the aluminum surface after corona discharge treatment and the condensation reaction has occurred between AlOOH and -Si-OH groups of the prehydrolyzed TES. Recently, secondary-ion mass spectrometry (SIMS) was used to directly detect the formation of Si-O-metal bonds at the interface between metals and silane films or silane-contained organic coatings. Guichenuy *et al*. [22] reported that in iron/polyamide coating system Si-O-Fe bond was detected at *m*/*z* = 99.907 at the coating interfaces in the case of incorporation of aminosilane into the powder formulation. The direct Si-O-Al bonding at aluminum/BTSE interface has also been identified via measurement of the depth profiles from the ratio of peak intensities at 71–70 amu by SIMS measurement [23]. The peak at 892 cm^−1^ is attributed to unreated silanol groups (-SiOH) [24] while the peak at 796 cm^−1^ is attributed to unhydrolyzed Si-OCH_3_ groups.

### 2.3. XPS Analysis 

**Figure 3:**
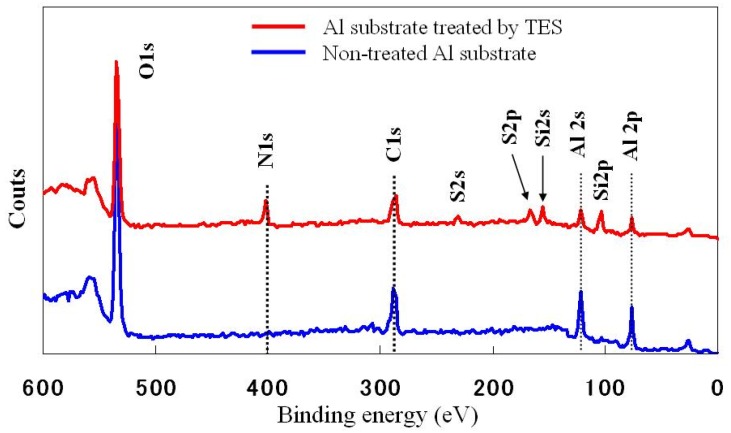
XPS wide scan spectra of the untreated and TES treated aluminum surfaces in 45 tilt degree. (X-ray anode: Al monochromated 2 nm filament; Aperture: 800 × 200 μm).

The XPS spectrum of untreated and TES treated aluminum substrates is shown in Figure 3. It is seen that only the peaks of Al2p (73.6 eV) and Al2s (119.7 eV) are observed, except for the peaks of C1s (285.0 eV), O1s (532.0 eV) for the untreated aluminum substrate. However, the peaks of N1s (400.7eV), S2s (228.2 eV), S2p (163.6 eV), Si2p (101.9 eV) and Si2s (151.8 eV) corresponding to the TES self-assembly monolayer are detected for the TES treated aluminum substrate. These results also confirmed the formation of a TES self-assembly monolayer on the metal surface. The atomic concentration for the relevant elements are summarized in Table 1. As in the case of TES treatment, there is an increased amount of Si, S and N on the aluminum surface, from 0% for the pure aluminum substrate to about 10.45% (Si2p), 3.00% (S2p) and 8.82% (N1s) after modification. From the results, it can be concluded that the Si-OH group of TES reacted with the polar groups on the pretreated aluminum surface to obtain a TES film.

### 2.4. Morphology (AFM) 

**Figure 4:**
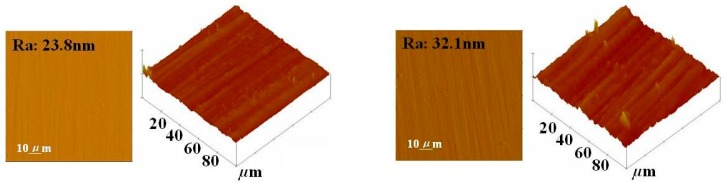
The AFM images before and after the formation of TES self-assembly monolayer on aluminum surface. **(a)** aluminum substrate untreated by TES (Left). **(b)** TES treated aluminum substrate (Right).

The changes in surface morphology of aluminum substrate before and after the formation of TES self-assembly monolayer were investigated by AFM. Figure 4(a) shows the AFM image of the non-treated aluminum surface. The surface is smooth, except for the presence of a few features which may be associated with surface contaminants and aluminum oxide. After cleaning with acetone, alkaline and corona discharge, followed by silanization with TES, changes in the surface roughness and morphology are observed, as shown in Figure 4(b). 

The morphology and roughness are important in controlling the modification and to obtain good adhesion properties. The surface is dominated by prominent features which may have resulted from the polarizing effects of alkaline and corona discharge, as well as from the silanization process.

From above distinctive changes in surface compositions and morphology after the formation of TES self-assembly monolayer on the baluminum plate, the molecular functionalities of metal and metal oxide surfaces can be redesigned through silanization with functional monomers to meet specific applications, such as adhesion promotion, corrosion protection, etc.

### 2.5. Adhesion Test 

**Figure 5:**
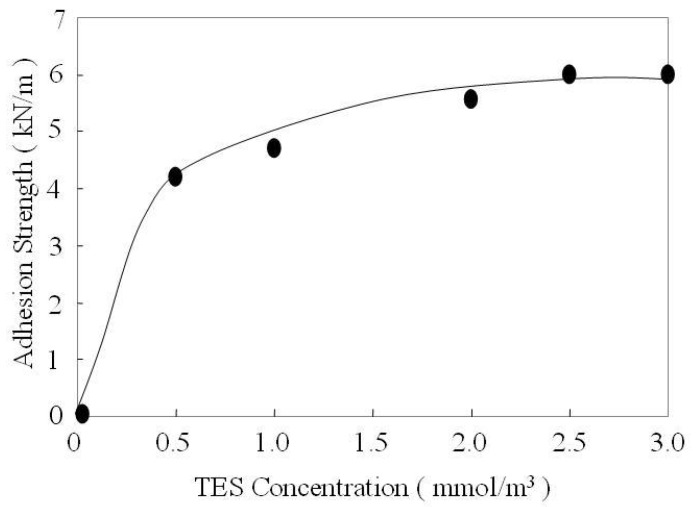
Effect of TES concentration on the adhesion strength between aluminum substrate and EPDM rubber.

The strong adherence of rubber to aluminum depends on the strength of molecular cross-linking at the different interfaces. One of these results from the formation of a covalent bond between the thiol group of TES self-assembly monolayer and the reactive groups of EPDM rubber. Therefore, the adherence force will depend on the number of bonds between the rubber and the aluminum surface. The effect of the TES self-assembly monolayer on the practical adhesion between aluminum and EPDM rubber are discussed from the aspects of TES concentration and cure temperature. Figure 5 shows the effect of TES concentration on adhesion strength between aluminum and EPDM rubber. Adhesion strength is measured for TES concentrations ranging from 0 to 3.0 mmol/m^3^. For the concentrations below 0.25 mmol/m^3^, it can be assumed that the adhesion strength is very low [25], and the adhesion strength grows steadily to 6.0 KN/m when the TES concentration increases to 2.5 mmol/m^3^. The fact that the adhesion strength is weak at low TES concentration is probably due to the lower coverage of cohesive silane layers on the aluminum surface. It will certainly lead to the formation of a covalent bond between aluminum and EPDM rubber. With the increasing TES concentration, the aluminum surface is almost covered by -Si-O-Al bonding. It makes that the bonding sites of aluminum surface and EPDM rubber are significantly increasing. As a result, adhesion strength has been improved. 

**Figure 6:**
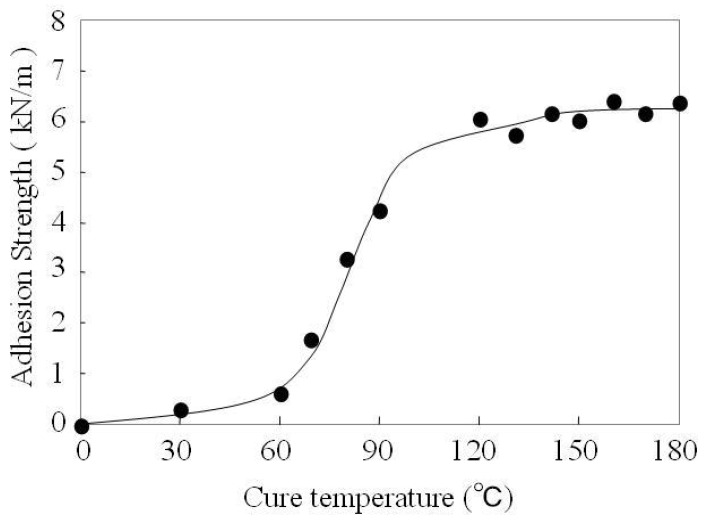
Effect of curing temperature for TES self-assembly monolayer on adhesion strength between aluminum substrate and EPDM rubber. (TES concentration: 2.5mmol/L).

The TES self-assembly monolayer must be cured to provide a good adherence to rubber. Here, the influence of this factor is also investigated. Figure 6 shows that the adhesion strength increases steadily with the curing temperature from room temperature (RT ≈ 20 °C) to about 180 °C. The fact that the adhesion strength is low at RT is probably due to the presence of several non-cohesive silane layers on the aluminum surface. At RT we can assume that only a few silanol groups are anchored to the surface through Al-O-Si- bonds; others are adsorbed through their thiol groups to AlOOH moieties and, above this layer, there are unbound randomly oriented silanes which contribute to the weak cohesion of the layer between EPDM rubber and aluminum. At high cure temperature pendant -SiOH groups react with other AlOOH moieties to form more covalent bands of Al-O-Si-, but also the thiol group of TES self-assembly monolayer may react with the reactive groups of the EPDM rubber to form covalent bonds. It can be assumed that the peroxide additive in the EPDM rubber is decomposed into the corresponding radical fragments and these radicals could initiate the unvulcanized sulfur mixed in the EPDM rubber to form the corresponding sulfur radicals. Then the sulfur radicals react with the thiol group of TES to obtain new covalent bonds.

**Figure 7:**
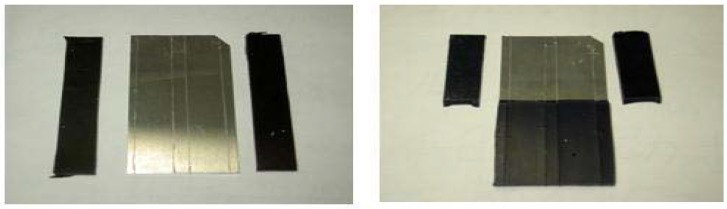
Photographs after T-peel test of untreated and TES treated aluminum substrate and EPDM rubber. **(a)** aluminum substrate untreated by TES (Left). **(b)** TES treated aluminum substrates (Right).

Figure 7a,b show the pictures after peel tests. Direct inspection shows that the aluminum substrate untreated by TES (Figure 7a) does not show any adhesive property to the EPDM rubber, while good and uniform adhesion is noted when TES self-assembly monolayer is used as the primer (Figure 7b). From the result, it can be concluded that TES may be used as effective adhesive between metal and rubber.

## 3. Experimental

### 3.1. Materials and Reagents 

Aluminum plates (0.1 × 300 × 300 mm) were purchased from the Nilaco Corporation Company. Aluminum samples for the experiments were prepared by cutting these larger plates into pieces of 0.1 × 30 × 60 mm size. EPDM rubber were provided by the Yokohama Rubber Co., Ltd. Additives are also present in this rubber: HAF carbon black (50 phr; phr = parts per hundred rubber), zinc oxide (5 phr), stearic acid (1 phr), *N*,*N*’-1,3-phenylenebismaleimide (2 phr), and dicumyl peroxide (DCP) (5 phr). 6-(3-Triethoxysilylpropylamino)-1,3,5-triazine-2,4-dithiol (TES) was prepared by reacting 6-(3-triethoxysilylpropylamino)-1,3,5-triazine-2,4-dichloride with NaSH. The compound is supplied by Sulfur Chemical Institute Inc. of Japan. The self-assembly solution consists of 2.5 mmol/dm^3^ silane monomer dissolved in *95/*5 (v/v) ethanol/distilled-water mixed solvent. The solution was then prehydrolyzed at room temperature for 24h (ageing time, *t*_ag_ = 24 h).

### 3.2. Surface Preparation

**Figure 8:**
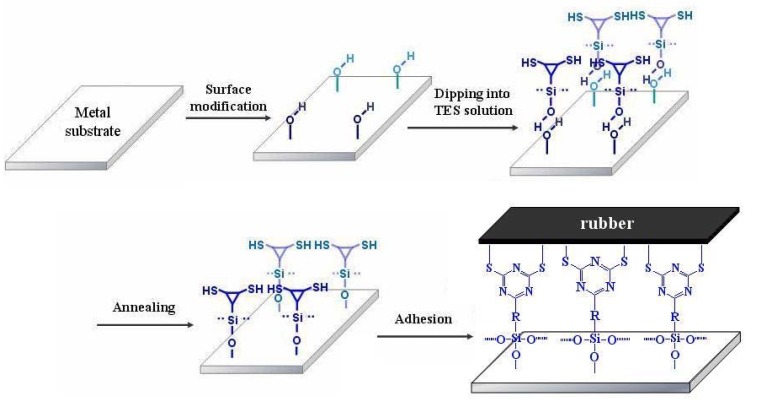
Model illustration of surface modification of the substrate by different steps.

Several steps are required and must be optimized to obtain the best adherence of aluminum substrate to EPDM rubber. First the aluminum surface must be thoroughly cleaned. Among the various chemical treatments available, we found that degreasing by ultrasonication in an acetone bath for 10 min, followed by treatment with an alkaline bath at 70 °C for 5min, rinsing with distilled water, drying by dryer, and then modification by corona discharge, were the most efficient cleaning process. An aluminum surface containing functional hydroxy groups was obtained by the process. The silanization of the aluminum surface is the second step: the aluminum plate is dipped into a prehydrolyzed containing 2.5 mmol/dm^3^ TES monomer ethanol-water solution for 5 min at room temperature. The TES self-assembly monolayer coated aluminum substrate is then dried in air, hung in an oven and cured at 160 °C for about 10 min. Next, the cured silanized aluminum plate is put in contact with EPDM rubber, followed by vulcanization for 30 min at 160 °C. The processes of corona discharge, TES treatment, curing and vulcanization are shown schematically in Figure 8.

### 3.3. Measurements

FT-IR spectra were measured at a resolution of 4 cm^−1^ by high-performance reflection absorption spectroscopy using a JASCO IR-5500 instrument. A reflection attachment was used at an incident angle of 80° together with a wire grid polarizer. Contact angles of pure water on the treated aluminum plates were determined using an Elma goniometer contact angle measuring apparatus (Elma-type G-1). Droplet diameter was controlled at 0.8 to 0.1 mm. X-ray photoelectron spectroscopy (XPS) was performed to determine the elemental composition of aluminum surface. Spectra were obtained using a ULVAC PHI-5600 spectrometer equipped with monochrome Al Kα radiation (1,486.6 eV). The pressure in the preparation chamber was less than 10^−^^7^ Torr and less than 4 × 10^−^^10^ Torr in the analysis chamber. Samples were examined over an 800 × 2,000 μm area, and potoelectron spectra are recorded with a take-off angle of 45°. The adhesion strength of TES self-assembly monolayer coated aluminum substrate to EPDM rubber was investigated by T-peel test using an autograph S-100 apparatus (Shimadsu Corporation).

## 4. Conclusions 

In this work, a TES self-assembly monolayer has been used to modify an aluminum surface. Significant morphological and chemical changes were produced by a series of pretreatments and TES modification. The results evidenced that the formed TES self-assembly monolayer was bound to aluminum surface through its ethoxysilyl function groups. The adhesion promotion performance between an aluminum substrate coated with a TES self-assembly monolayer and EPDM rubber was proven. Considerable adhesion strength was obtained when the aluminum was silanized with 2.5 mmol/dm^3^ TES in ethanol–water solution, cured at 160 °C and vulcanized with EPDM rubber at 160 °C for 30 min. By increasing the curing temperature from room temperature to about 180 °C, adhesion strength was improved steadily. It may be considered that the TES self-assembly monolayer is an effective, interesting and environmental friendly method to modify metal surfaces and improve adhesion performance for technical applications.

## Figures and Tables

**Table 1:** Atomic concentrations of TES treated and non-treated aluminum surfaces.

*Aluminum* *Surface*	*C1s* *(%)*	*N1s* *(%)*	*S2p* *(%)*	*Si2p* *(%)*	*Al2p* *(%)*	*O1s* *(%)*
**Al non-treated by TES**	22.24	-	-	-	27.36	50.40
**Al treated by TES**	22.86	8.82	3.00	10.45	11.67	43.2
